# Rural-Urban Disparities in Survival Among Patients With Malignant Primary Osseous Neoplasms of the Spine and Sacropelvis: A Population-Based Cohort Study

**DOI:** 10.7759/cureus.115999

**Published:** 2026-09-09

**Authors:** Graydon Dietrich, Jerard Tambunan, Elijah Haynal, Houston Macintyre, David Campbell, Dewayne Weaver

**Affiliations:** 1 School of Medicine, Loma Linda University, Loma Linda, USA; 2 Orthopaedic Surgery, St. Joseph Regional Medical Center, Lewiston, USA

**Keywords:** primary bone tumors, rural health, seer database, socioeconomic disparities, spinal neoplasms, survival analysis

## Abstract

Background

Malignant primary osseous neoplasms of the spine and sacropelvis are rare tumors whose outcomes rely heavily on specialized, guideline-driven care, which is concentrated in metropolitan referral centers. Investigations of rurality in this population have been limited by single-histology cohorts, small numbers of rural patients, and unadjusted comparisons, and none have weighed geographic disadvantage directly against the socioeconomic conditions with which it coexists. This study aimed to determine whether rural residence is independently associated with overall survival and cause-specific survival, and secondarily whether county-level median household income is independently associated with survival in the same models.

Methodology

This was a retrospective, population-based cohort study using the Surveillance, Epidemiology, and End Results (SEER) database from 2000 to 2023. Patients with microscopically confirmed malignant chordoma, chondrosarcoma, osteosarcoma, or Ewing sarcoma of the vertebral column or the pelvic bones, sacrum, or coccyx with known survival time were included. Rurality was defined using SEER county attributes, with nonmetropolitan counties classified as rural. Multivariable Cox proportional hazards models for overall survival and cause-specific survival were adjusted for demographic, socioeconomic, tumor, and treatment characteristics, with time-based interaction terms added where Schoenfeld residuals indicated violation of the proportional hazards assumption.

Results

Of 3,631 patients (median age = 51 years; 59% male), 3,255 (90%) resided in urban counties and 376 (10%) in rural counties. Rural residence was concentrated in low-income counties (54% below $60,000 vs. 4.1% of urban patients). Rurality was not independently associated with overall survival (hazard ratio (HR) = 0.95, 95% CI (confidence interval) = 0.82-1.09; p = 0.449) or cause-specific survival (HR = 0.94, 95% CI = 0.80-1.11; p = 0.457). County income showed a graded association with overall survival relative to counties below $60,000, with HRs of 0.82 ($80,000-$100,000), 0.72 ($100,000-$120,000), and 0.68 (>$120,000), all with p-values <0.001, and no difference for $60,000-$80,000 (HR = 1.01). A similar gradient was observed for cause-specific survival. Surgery was associated with lower mortality (overall survival: HR = 0.76; cause-specific survival: HR = 0.80; both p < 0.001), whereas radiotherapy was not. Unadjusted differences by rurality in stage at presentation and treatment receipt were small.

Conclusions

Rural residence conferred no independent survival disadvantage once socioeconomic and clinical factors were accounted for, whereas county-level income remained independently associated with mortality in a graded fashion. Because rural residence and low county income overlap heavily in this cohort, the survival signal appears to be carried by socioeconomic context rather than by geography. These tumors are rare, are not amenable to screening, and are largely managed at specialized referral centers, which may explain the absence of the rural disadvantage described for screen-detectable cancers. Identifying at-risk patients by geography alone may overlook socioeconomically disadvantaged patients in urban counties, and direct assessment of financial and access-related barriers may be more informative than rural-urban classification.

## Introduction

Primary malignant neoplasms of the osseous spine constitute a small percentage of all primary bone cancers [1,2]. These rare neoplasms typically arise in the mobile spine or sacrum, where they are surrounded by critical anatomic structures, making resection difficult and prognosis poor [2,3]. Treatment of primary bone cancers varies by histologic subtype but generally consists of wide en bloc resection with neoadjuvant chemotherapy and radiotherapy [4,5]. For surgeons, achieving adequate margins in the sacrum and spine is technically challenging and carries a high rate of complications [4,6].

Patient outcomes in these tumors rely on the adequacy of surgical resection, and treatment at high-volume academic or referral centers has been associated with higher rates of survival in patients with primary malignant bone tumors of the osseous spine [7]. This advantage does not appear to result from differences in surgeon technical skill, as margin status and adjuvant therapy were similar between high- and low-volume centers [7]. A study of patients with primary tumors of the mobile spine found that those treated at a National Cancer Institute (NCI)-designated cancer center were more likely to receive guideline-concordant treatment, which, in turn, was associated with lower mortality [8]. Together, these findings suggest that outcomes in primary spinal tumors are shaped not only by tumor biology but also by access to specialized, guideline-concordant care.

Access to high-volume academic or referral centers is not distributed evenly. Such institutions are predominantly located in metropolitan areas, and rural patients must often travel long distances to obtain specialized care. Greater distance to care has been associated with more advanced stage at diagnosis and less appropriate treatment across a range of cancers [9]. Rural residence itself also carries a measurable survival disadvantage; a recent meta-analysis found that rural patients had 15% lower odds of overall survival and 10% lower odds of cancer-specific survival, with the disadvantage worsening at greater degrees of remoteness [10]. However, distance is not the only disadvantage these patients face. Although the current evidence is limited, socioeconomic status has also been implicated in outcomes. Patients with lower income often appear to have worse survival on unadjusted comparison, but these differences tend to attenuate once other factors are accounted for. Ganga et al. reported that neither Medicaid coverage nor poverty level was associated with survival once other patient characteristics were considered [11]. Additionally, lower income, while associated with worse survival in simpler comparisons, has not reliably held up in more rigorous models [12,13]. Rurality and low income are difficult to separate because the two often overlap. In a recent chondrosarcoma Surveillance, Epidemiology, and End Results (SEER) study, 58% of rural households fell in the lowest income stratum compared with 3.4% of urban households [13].

In previous studies examining rurality among populations with primary osseous spinal neoplasms, findings have been inconsistent and methodologically limited. Lin et al. reported that rural residence was associated with lower mortality on Cox analysis in chondrosarcoma [13], whereas a National Cancer Database study of chordoma of the spine reported unadjusted lower survival among rural patients, in a cohort that included only 20 rural patients [12]. A separate SEER analysis of sacral and vertebral column chordoma reported no significant difference in survival by urban county background [14]. The limitations of these prior studies included single-histology cohorts, unadjusted comparisons, limited cohort size, and models that adjusted for rurality without ever reporting its effect. No study to date has tested rurality as an independent predictor of survival across all malignant primary osseous spinal and sacropelvic neoplasms or weighed geographic disadvantage directly against the socioeconomic conditions with which it coincides. The primary aim of the study was to determine whether rural residence is independently associated with overall survival and cancer-specific survival in patients with malignant primary osseous spinal neoplasms. Secondarily, we examined whether county-level income was independently associated with survival in the same models, and described stage at presentation and treatment receipt by rurality.

## Materials and methods

This was a retrospective, population-based cohort study using the SEER database, including diagnoses occurring from 2000 to 2023. All database access was conducted using the NCI SEER*Stat software (National Cancer Institute, Bethesda, MD), querying the November 2025 database submission. Given the de-identified nature of the SEER database, this study received an exempt status from the authors’ institutional review board at Loma Linda University (approval number: 5260445). Tumors were selected using International Classification of Diseases for Oncology, Third Edition (ICD-O-3) codes: chordoma (9370/3, 9371/3, 9372/3), chondrosarcoma (9220/3, 9221/3, 9222/3, 9230/3, 9231/3, 9240/3-9243/3), osteosarcoma (9180/3-9187/3, 9192/3-9194/3), and Ewing sarcoma (9260/3). Anatomic location was limited by ICD-O-3 code to the vertebral column (C41.2) and pelvic bones, sacrum, or coccyx (C41.4). Only tumors of malignant behavior (/3) were included, and all cases required microscopic confirmation of diagnosis and a known survival time.

Variables obtained for each record included overall and cause-specific survival in months, age, sex, race, Spanish-Hispanic-Latino origin, median county income, county rurality, tumor extension (local, regional, distant), tumor classification, tumor location, surgical treatment, and radiotherapy. The exposure of interest was residence within a rural county, as recorded in the SEER county attributes database. Median county income brackets were provided by the SEER database and inflation-adjusted to 2024 levels. Counties classified as metropolitan (rural-urban continuum codes 1-3) were categorized as urban regardless of population, while nonmetropolitan counties were categorized as rural (rural-urban continuum codes 4-9). The primary outcomes were overall survival and cause-specific survival, recorded in the SEER database as vital status and cause-specific death, respectively. Cause-specific survival was defined as the time from diagnosis to death attributable to the study malignancy, as classified by the SEER cause-specific death classification variable. Patients who died of causes other than the study malignancy, or who remained alive at last follow-up, were censored at the time of death or last contact, respectively. This differs from overall survival, in which death from any cause is treated as the event of interest. Surgery and radiotherapy were coded as fixed baseline covariates using SEER first-course-of-treatment fields because high missingness in the time-to-treatment data precluded modeling treatment as time-varying covariates. Missing data were coded as “unknown” levels within covariates rather than excluding them from analysis.

All statistical analyses were performed using R version 4.5.2 (R Foundation for Statistical Computing, Vienna, Austria) via RStudio version 2023.6.0.421 (Posit, PBC, Boston, MA). Cox proportional hazards modeling was used for overall survival and cause-specific survival analyses. Median county income was modelled as categorical for main analyses, and additional survival analyses utilizing ordinal modeling for median county income were conducted to discuss potential graded effects. Time-based interaction terms were included as needed when examination of the Schoenfeld residuals indicated significant violations of the proportional hazards assumption. In the overall survival model, time-based interaction terms were required for age, tumor extension, and tumor classification. In the cause-specific survival model, they were required for tumor extension and tumor classification. Interaction terms between rurality and county-level income and prescribed patient subgroups were also tested to assess effect modification but were not found to be significant. Multicollinearity among covariates was assessed using variance inflation factors (VIFs), with values >5 considered indicative of problematic collinearity. As a sensitivity analysis to address the potential influence of competing risks on the cause-specific survival findings, a Fine-Gray subdistribution hazard model was fit with non-cancer death treated as a competing event and deaths of unknown cause classified as cancer-specific deaths, consistent with the primary cause-specific Cox model. Proportional hazards for the subdistribution hazard model were assessed using Schoenfeld residuals; because several covariates showed evidence of non-proportionality, hazard ratios (HRs) are reported only for the two exposures of primary interest, rurality and county-level income. A small number of patients belonging to the American Indian/Alaska Native SEER-defined race category were omitted due to low counts, creating concerns for statistical instability and for compliance with SEER data suppression regulations. The topmost income bracket is collapsed to >$100,000 to allow for compliance with SEER data suppression regulations. The threshold for statistical significance was set at alpha = 0.05 for all analyses.

## Results

Baseline characteristics

A total of 3,631 patients with malignant primary osseous spinal neoplasms were included in the analysis, with demographics, baseline characteristics, and covariate missingness shown in Table 1. The median age of the cohort was 51 years (interquartile range = 27-67), and the cohort was predominantly male (2,138; 59%). Patients were predominantly White (3,091; 85%) and non-Spanish-Hispanic-Latino (2,993; 82%). Approximately half of the patients were unmarried (1,769; 49%). The majority of patients lived in urban counties (3,255; 90%), with 376 (10%) patients residing in rural counties. County-level median household income distribution differed substantially between rural and urban groups. Overall, 337 (9.3%) patients lived in counties with a median household income below $60,000, 1,093 (30%) in counties of $60,000-$80,000, 1,172 (32%) in counties of $80,000-$100,000, and 1,029 (28%) in counties above $100,000. Among rural patients, 203 of 376 (54%) lived in the lowest income group compared with 134 of 3,255 (4.1%) urban patients, while only nine (2.4%) rural patients resided in counties with a median income above $100,000 compared with 1,020 (31%) urban patients. Chondrosarcoma (32%), chordoma (29%), Ewing sarcoma (19%), and osteosarcoma (20%) were all substantially represented, and the majority of tumors were located in the pelvis (71%). The majority of patients received some form of surgical treatment (64%), while a minority received radiotherapy (39%).

**Table 1:** Cohort demographics and baseline characteristics. Continuous variables are presented as median (Q1, Q3), and categorical variables as n (%). The highest income bracket is collapsed for compliance with SEER data suppression regulations. STR = subtotal resection; GTR = gross total resection; SEER = Surveillance, Epidemiology, and End Results

Variable	County rurality		
	All	Urban	Rural
	N = 3,631	N = 3,255	N = 376
Age (years)	51 (27, 67)	51 (28, 67)	52 (21, 68)
Sex			
Female	1,493 (41%)	1,326 (41%)	167 (44%)
Male	2,138 (59%)	1,929 (59%)	209 (56%)
Race			
White	3,091 (85%)	2,745 (84%)	346 (92%)
Asian or Pacific Islander	298 (8.2%)	286 (8.8%)	12 (3.2%)
Black	242 (6.7%)	224 (6.9%)	18 (4.8%)
Ethnicity			
Non-Spanish-Hispanic-Latino	2,993 (82%)	2,647 (81%)	346 (92%)
Spanish-Hispanic-Latino	638 (18%)	608 (19%)	30 (8.0%)
Median county income			
<$60,000	337 (9.3%)	134 (4.1%)	203 (54%)
60,000–80,000	1,093 (30%)	956 (29%)	137 (36%)
80,000–100,000	1,172 (32%)	1,145 (35%)	27 (7.2%)
>$100,000	1,029 (28%)	1,020 (31%)	9 (2.4%)
Marital status			
Unmarried	1,769 (49%)	1,579 (49%)	190 (51%)
Married	1,716 (47%)	1,546 (47%)	170 (45%)
Unknown	146 (4.0%)	130 (4.0%)	16 (4.3%)
Tumor extension			
Localized	1,204 (33%)	1,086 (33%)	118 (31%)
Regional	1,254 (35%)	1,119 (34%)	135 (36%)
Distant	580 (16%)	510 (16%)	70 (19%)
Unknown	593 (16%)	540 (17%)	53 (14%)
Tumor classification			
Chondrosarcoma	1,159 (32%)	1,044 (32%)	115 (31%)
Chordoma	1,042 (29%)	958 (29%)	84 (22%)
Ewing sarcoma	696 (19%)	610 (19%)	86 (23%)
Osteosarcoma	734 (20%)	643 (20%)	91 (24%)
Tumor location			
Pelvis	2,563 (71%)	2,303 (71%)	260 (69%)
Spinal column	1,068 (29%)	952 (29%)	116 (31%)
Surgery			
No surgery	1,325 (36%)	1,173 (36%)	152 (40%)
Biopsy only	96 (2.6%)	90 (2.8%)	6 (1.6%)
STR	9 (0.2%)	9 (0.3%)	0 (0%)
GTR	908 (25%)	821 (25%)	87 (23%)
Surgically treated, extent unknown	1,293 (36%)	1,162 (36%)	131 (35%)
Radiotherapy			
No/Unknown	2,204 (61%)	1,968 (60%)	236 (63%)
Radiation	1,427 (39%)	1,287 (40%)	140 (37%)

Overall survival

Hazard ratios (HRs) from the multivariable Cox proportional hazards model for overall survival are presented in Figure 1. No covariates demonstrated evidence of problematic multicollinearity (all VIF < 5). County classification as rural or urban was not associated with a statistically significant difference in overall survival (HR = 0.95, 95% confidence interval (CI) = 0.82-1.09; p = 0.449). Median county income showed a consistent, graded association with overall survival. Relative to counties with a median income below $60,000, mortality was progressively lower in counties with median incomes of $80,000 to $100,000 (HR = 0.82, 95% CI = 0.74-0.90; p < 0.001), $100,000 to $120,000 (HR = 0.72, 95% CI = 0.64-0.82; p < 0.001), and above $120,000 (HR = 0.68, 95% CI = 0.57-0.80; p < 0.001); the $60,000 to $80,000 stratum did not differ from the reference (HR = 1.01, 95% CI = 0.91-1.11; p = 0.900). Modeling median county income as an ordinal variable, each one-category increase in income was associated with a 12.2% decrease in the hazard of death (HR = 0.879, 95% CI = 0.842-0.916; p for trend < 0.001).

**Figure 1:**
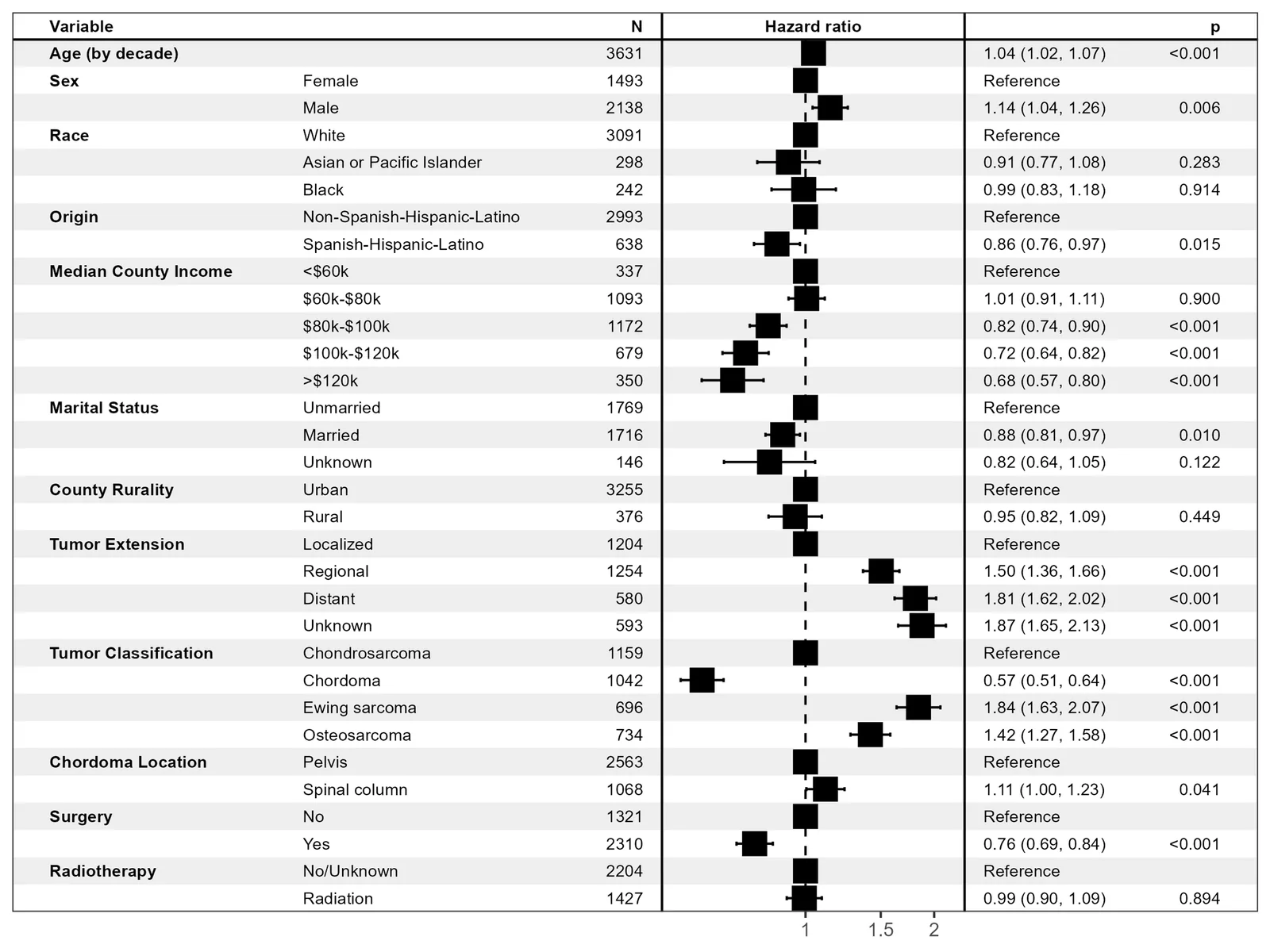
Forest plot of multivariable Cox proportional hazards model for overall survival. Time-based interaction terms were included for age, tumor extension, and tumor classification.

Male sex (HR = 1.14, 95% CI = 1.04-1.26; p = 0.006) was associated with higher mortality, whereas Spanish-Hispanic-Latino origin (HR = 0.86, 95% CI = 0.76-0.97; p = 0.015) and married status (HR = 0.88, 95% CI = 0.81-0.97; p = 0.010) were associated with lower mortality. Race was not associated with overall survival (Asian or Pacific Islander: HR = 0.91, 95% CI = 0.77-1.08, p = 0.283; Black: HR = 0.99, 95% CI = 0.83-1.18, p = 0.914). The HR for age (per decade) was 1.04 (95% CI = 1.02-1.07; p < 0.001).

Compared with localized disease, mortality was higher for regional (HR = 1.50, 95% CI = 1.36-1.66; p < 0.001), distant (HR = 1.81, 95% CI = 1.62-2.02; p < 0.001), and unknown extension (HR = 1.87, 95% CI = 1.65-2.13; p < 0.001). Relative to chondrosarcoma, chordoma carried a substantially lower hazard of death (HR = 0.57, 95% CI = 0.51-0.64; p < 0.001), while Ewing sarcoma (HR = 1.84, 95% CI = 1.63-2.07; p < 0.001) and osteosarcoma (HR = 1.42, 95% CI = 1.27-1.58; p < 0.001) carried higher hazards. Tumors of the spinal column carried a modestly higher hazard than those of the pelvis (HR = 1.11, 95% CI = 1.00-1.23; p = 0.041). Receipt of surgical treatment was associated with lower mortality (HR = 0.76, 95% CI = 0.69-0.84; p < 0.001), whereas receipt of radiotherapy was not (HR = 0.99, 95% CI = 0.90-1.09; p = 0.894).

Time-interaction terms indicated that these effects were not constant over follow-up: the elevated hazard associated with all levels of tumor extension diminished substantially with increasing follow-up time (all p < 0.001), the protective effect of chordoma relative to chondrosarcoma attenuated over time (interaction HR 1.007 per unit time, p < 0.001), and the elevated hazard associated with Ewing sarcoma modestly decreased with longer follow-up (interaction HR = 0.997, p = 0.025); osteosarcoma showed no significant time-dependent change (p = 0.719). The hazard associated with age increased modestly with longer follow-up (interaction HR = 1.002 per decade per unit time, p < 0.001).

Cause-specific survival

HRs from the multivariable Cox model for cause-specific survival are presented in Figure 2. No covariates demonstrated evidence of problematic multicollinearity (all VIF < 5). County rurality was not associated with a statistically significant difference in cause-specific survival (HR = 0.94, 95% CI = 0.80-1.11; p = 0.457). The income gradient observed for overall survival persisted for cause-specific survival, with lower cancer-specific mortality in counties with median incomes of $80,000 to $100,000 (HR = 0.84, 95% CI = 0.75-0.95; p = 0.003), $100,000 to $120,000 (HR = 0.72, 95% CI = 0.62-0.83; p < 0.001), and above $120,000 (HR = 0.74, 95% CI = 0.61-0.89; p = 0.002) relative to counties below $60,000. Modeling median county income as an ordinal variable, each one-category increase in income was associated with an 11.1% decrease in the hazard of death (HR = 0.890, 95% CI = 0.848-0.934; p for trend < 0.001).

**Figure 2:**
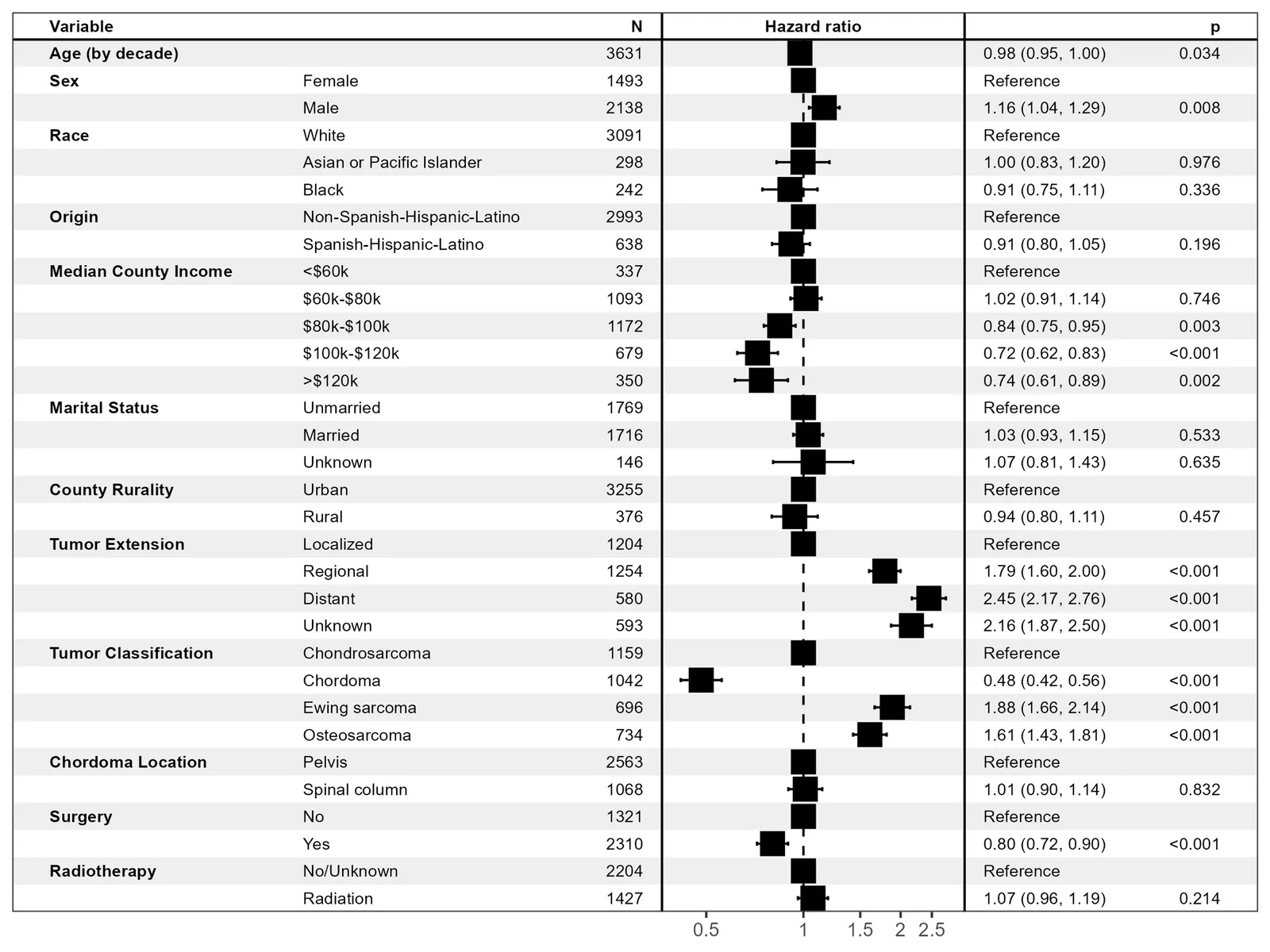
Forest plot of multivariable Cox proportional hazards model for cause-specific survival. Time-based interaction terms were included for tumor extension and tumor classification.

Male sex remained associated with higher cancer-specific mortality (HR = 1.16, 95% CI = 1.04-1.29; p = 0.008). In contrast to the overall survival model, neither Spanish-Hispanic-Latino origin (HR = 0.91, 95% CI = 0.80-1.05; p = 0.196) nor married status (HR = 1.03, 95% CI = 0.93-1.15; p = 0.533) was associated with cause-specific survival, and race again showed no association. The HR for age (per decade) was 0.98 (95% CI = 0.95-1.00; p = 0.034).

Tumor extension carried hazards of 1.79 (95% CI = 1.60-2.00; p < 0.001) for regional, 2.45 (95% CI = 2.17-2.76; p < 0.001) for distant, and 2.16 (95% CI = 1.87-2.50; p < 0.001) for unknown extension relative to localized disease. Relative to chondrosarcoma, chordoma again carried a lower hazard (HR = 0.48, 95% CI = 0.42-0.56; p < 0.001), while Ewing sarcoma (HR = 1.88, 95% CI = 1.66-2.14; p < 0.001) and osteosarcoma (HR = 1.61, 95% CI = 1.43-1.81; p < 0.001) carried higher hazards. Receipt of surgical treatment demonstrated lower hazard (HR = 0.80, 95% CI = 0.72-0.90; p < 0.001), while radiotherapy (HR = 1.07, 95% CI = 0.96-1.19; p = 0.214) and tumor location (spinal column: HR = 1.01, 95% CI = 0.90-1.14; p = 0.832) were not associated with differences in cause-specific survival.

Time-interaction terms again indicated that these effects were not constant over follow-up: the elevated hazard associated with all levels of tumor extension diminished substantially with increasing follow-up time (all p < 0.001), the protective effect of chordoma relative to chondrosarcoma attenuated over time (interaction HR = 1.008 per unit time, p < 0.001), and the elevated hazard associated with Ewing sarcoma modestly decreased with longer follow-up (interaction HR = 0.993, p < 0.001); osteosarcoma showed no significant time-dependent change (p = 0.719).

As a sensitivity analysis addressing competing risks, a Fine-Gray subdistribution hazard model was fit with non-cancer death treated as a competing event. Because several covariates in this model showed evidence of non-proportional subdistribution hazards on Schoenfeld residual testing, HRs are reported only for rurality and county income, the primary exposures of interest, for which the proportional hazards assumption was met. Rurality remained non-significantly associated with the subdistribution hazard of cancer-specific death (HR = 1.03, 95% CI = 0.86-1.25; p = 0.731), consistent with the cause-specific Cox model. The non-monotonic association with county income was also preserved, with the lowest subdistribution hazard again observed in the $100,000-$120,000 stratum (HR = 0.77, 95% CI = 0.61-0.97; p = 0.028) relative to counties below $60,000; HRs for the $60,000-$80,000 (HR = 0.98, 95% CI = 0.80-1.20; p = 0.854), $80,000-$100,000 (HR = 0.95, 95% CI = 0.77-1.18; p = 0.648), and >$120,000 (HR = 0.83, 95% CI = 0.64-1.08; p = 0.168) strata were not significant.

## Discussion

There was no statistically significant evidence of an independent association between rural residence and either overall or cancer-specific survival after adjustment for demographic, socioeconomic, tumor, and treatment characteristics in this cohort of 3,631 patients. For overall survival, the HR associated with rural residence was 0.95 (95% CI = 0.82-1.09; p = 0.449), and the corresponding HR for cancer-specific survival was similar at 0.94 (95% CI = 0.80-1.11; p = 0.457). This finding directly addresses the primary study objective.

In contrast, county-level income was independently associated with survival, with progressively lower mortality observed beginning at median county household incomes of $80,000. Using patients living in counties with a median household income below $60,000 as the reference group, there was no meaningful difference in mortality among those living in counties with median incomes of $60,000-$80,000 (HR = 1.01, 95% CI = 0.91-1.11). Progressively lower hazards were observed among patients residing in counties with median incomes of $80,000-$100,000 (HR = 0.82, 95% CI = 0.74-0.90), $100,000-$120,000 (HR = 0.72, 95% CI = 0.64-0.82), and greater than $120,000 (HR = 0.68, 95% CI = 0.57-0.80). Thus, while rural versus urban residence was not independently associated with survival, county-level socioeconomic status remained associated with mortality after simultaneous adjustment for rurality and clinical characteristics.

Several clinical factors were also independently associated with survival. Greater tumor extension was associated with higher mortality, while receipt of surgery was associated with lower mortality (HR = 0.76, 95% CI = 0.69-0.84; p < 0.001). Histologic subtype remained prognostic, consistent with the differing natural histories of chordoma, chondrosarcoma, Ewing sarcoma, and osteosarcoma [2,3]. These findings indicate that tumor biology, disease extent, and definitive local treatment remained associated with outcome in the adjusted models.

Radiotherapy was not independently associated with overall survival in the adjusted model. This finding should not be interpreted as evidence that radiotherapy lacks clinical benefit. The use of radiotherapy depends on histology, disease extent, resectability, surgical margins, radiation modality, and treatment intent, several of which cannot be adequately assessed in the present dataset. The lack of an independent association should therefore be interpreted cautiously given the heterogeneity in indications for radiotherapy and the absence of several treatment-specific variables in SEER. Moreover, radiotherapy may be used for other reasons such as local control, to address surgical margins, or to reduce recurrence risk rather than primarily to improve overall survival, which are outcomes that are not adequately captured in SEER [4].

Descriptive comparisons of the cohort showed modest differences in stage at presentation and receipt of treatment between rural and urban patients. Rural patients had a slightly higher proportion of distant disease at diagnosis (19% vs. 16%), were slightly more often categorized as receiving no surgery (40% vs. 36%), and were slightly less likely to receive radiotherapy (37% vs. 40%). Because these comparisons were unadjusted, they should not be interpreted as independent rural disparities and cannot establish whether stage or treatment differs by rurality after accounting for other factors.

Rural disparities in cancer outcomes have been documented, although the magnitude varies across cancer types and by degree of geographic remoteness. In a systematic review and meta-analysis, Marshall et al. reported that rural patients had 15% lower odds of overall survival and 10% lower odds of cancer-specific survival compared with urban patients after adjustment for clinical and demographic factors [10]. The strength of this association was not consistent across all malignancies. This variation is relevant to the present study, in which rural residence was not independently associated with either overall or cancer-specific survival.

Prior studies of primary spinal tumors have reported mixed findings. Battistin et al. examined 1,769 patients with spinal chordoma in the National Cancer Database and reported lower unadjusted survival among patients living in rural areas, along with associations between survival and socioeconomic characteristics, insurance status, treatment setting, and access to surgical care [12]. Because their analysis was limited to chordoma and focused on access-related factors, these findings are not directly comparable with the present study.

Pan et al. evaluated 808 patients with primary spinal chordoma using SEER data from 1973 to 2014 and found no significant association between urban county background and overall survival (p = 0.72) [14]. Their principal independent predictors were older age, distant disease, and absence of surgery. Although Pan et al. did not report a rurality-specific HR directly comparable with ours, their lack of a significant geographic survival association is consistent with the present findings.

Ganga et al. evaluated survival among patients with primary spinal osseous malignant tumors according to Medicaid status and neighborhood poverty. Medicaid patients had significantly worse unadjusted five-year survival (52.0% vs. 67.2%) and were less likely to receive surgery; minority individuals on Medicaid had an elevated risk of cancer-specific mortality in adjusted analyses [11]. These results differ in emphasis from the present study, in which county-level income remained independently associated with survival and showed a graded relationship with mortality. Differences may reflect cohort size, the socioeconomic measures used, or residual confounding.

Access to specialized treatment may remain relevant in these rare malignancies. Stroud et al. found that receipt of standard treatment protocols was associated with lower mortality among patients with primary osseous malignancies of the mobile spine, and treatment at an NCI-designated cancer center increased the likelihood of receiving standard therapy. Public insurance was associated with higher mortality compared with private insurance [8]. Lazarides et al. reported that higher facility volume was independently associated with improved survival among patients with primary malignant bone tumors of the vertebral column [7]. These studies suggest that access to specialized care may be more clinically relevant than geographic residence alone.

To our knowledge, the present study expands on this literature by analyzing the largest population-based cohort focused specifically on malignant primary osseous tumors of the spine and sacropelvis, including chordoma, chondrosarcoma, osteosarcoma, and Ewing sarcoma, while evaluating rurality and county-level income simultaneously within the same adjusted survival models.

Although our results found no statistically significant evidence of rural association, this should not be interpreted as evidence that rural patients face no disadvantages. Rural patients frequently encounter barriers to cancer care, including transportation limitations, longer travel distances, and reduced access to specialized services and guideline-concordant treatment [15]. The absence of a statistically significant rural survival disadvantage may partly reflect the close overlap between rural residence and low income. In our cohort, 54% of rural patients resided in counties with a median household income below $60,000, compared with only 4.1% of urban patients. This close overlap means analyses that do not account for income may mistake a socioeconomic disadvantage for a geographic one. In our data, however, rural residence was not associated with survival regardless of income, while county-level income showed an independent, graded association. This pattern suggests that socioeconomic context may be more informative than rurality alone in this population, although the substantial overlap between these variables limits the extent to which their independent contributions can be disentangled. The literature further indicates that rural survival disadvantages are not universal across cancer types. The rural survival disadvantage is more pronounced for common cancers such as breast and colorectal cancer, which are detectable through early screening where earlier diagnosis meaningfully improves survival [10]. For these common cancers, reduced access to screening and routine care in rural areas leads to delayed diagnosis and suboptimal outcomes [15]. In contrast, primary osseous neoplasms of the spine and sacropelvis differ from these cancers in that they are rare, cannot be screened for, and are managed almost exclusively at specialized referral centers, whether or not a patient lives in a rural or urban setting [7,8]. These differences may help explain why a rural survival disadvantage was not detected in this cohort, although the relatively small number of rural patients may also have limited statistical precision.

The graded association between county-level income and mortality was one of the most notable findings of this study. Mortality progressively decreased among patients residing in counties with median household incomes of $80,000-$100,000, $100,000-$120,000, and greater than $120,000, while there was essentially no difference between the two lowest income strata. A similar pattern was observed for cancer-specific survival. In contrast, rural residence was not independently associated with either outcome. Together, these findings suggest that county-level income may be a more sensitive marker of socioeconomic disadvantage than rurality alone. A binary rural-versus-urban classification may obscure substantial variation in resources within both settings, as urban residence does not necessarily imply adequate access to specialized cancer care.

County-level income may also reflect barriers that are not captured by geographic classification alone. Greater financial resources may facilitate access to higher-quality insurance coverage, travel and lodging near tertiary referral centers, time away from work, caregiver support, second opinions, treatment adherence, rehabilitation, and continuity of follow-up. These considerations may be particularly important for rare primary osseous tumors of the spine and sacropelvis, for which patients frequently require referral to specialized, high-volume centers regardless of whether they reside in a rural or urban area. Although these mechanisms could not be directly evaluated in SEER, they provide plausible explanations for why socioeconomic context may remain associated with survival even in the absence of an independent rural effect.

Our findings differ somewhat from those of Ganga et al., who examined Medicaid status and neighborhood poverty and found that socioeconomic measures did not uniformly remain independently associated with survival after adjustment [11]. Differences in the socioeconomic measures used, cohort composition, and statistical power may contribute to these differing findings. Importantly, geographic and socioeconomic disadvantage remain closely intertwined in the present cohort. More than half of rural patients resided in counties with median household incomes below $60,000 compared with only 4.1% of urban patients. Thus, although the survival association was more strongly observed with socioeconomic context than with geographic classification when both were modeled simultaneously, these findings do not establish that income explains or mediates the relationship between rurality and survival. Rurality and income remain highly related and may partially compete statistically within multivariable models. Furthermore, county-level income is an ecological measure rather than an estimate of individual patient income, and substantial socioeconomic heterogeneity may exist among patients residing within the same county.

Although rural patients had a slightly higher proportion of distant disease at diagnosis, they also had a lower proportion of unknown-stage disease (14% vs. 17%). This raises the possibility that the difference reflects more complete staging, rather than later diagnosis. Similarly, while a higher proportion of rural patients were categorized as receiving no surgery, the rates of gross total resection were comparable between the two groups (23% vs. 25%).

Importantly, none of these unadjusted differences corresponded to a difference in overall or cancer-specific survival. This pattern is in line with the central finding of this study. The small geographic differences that were observed in the descriptive comparisons did not translate into a survival disadvantage once clinical and socioeconomic factors were considered. 

Because these comparisons were unadjusted, and stage and treatment were not modeled as outcomes, they cannot establish whether rurality independently influences patients’ stage at presentation or treatment receipt. Formal multivariable analysis would be needed to answer this question directly. Nevertheless, the absence of a corresponding survival difference suggests that any such geographic differences are unlikely to be clinically substantial in this population.

Our findings suggest that identifying patients at risk based on rural residence alone may overlook vulnerable patients living in lower-income urban areas. Because county-level income remained associated with survival while rurality did not, health systems may benefit from assessing socioeconomic barriers directly rather than relying on geographic classification as a proxy for access. Such assessments could include insurance status, transportation needs, employment constraints, financial strain, housing needs during treatment, and caregiver availability. This may be especially important for patients with rare spinal and sacropelvic tumors, which often require costly, multidisciplinary care. Early involvement of financial counselors, social workers, insurance navigators, and patient-assistance programs could help address barriers related to prior authorizations, out-of-network referrals, copayments, rehabilitation expenses, and time away from work.

Access to specialized care also carries substantial logistical costs. Because these tumors are frequently managed at tertiary or high-volume referral centers, patients may need to travel considerable distances even when they reside in urban counties. Transportation vouchers, temporary lodging, parking assistance, and travel reimbursement may reduce the financial burden associated with obtaining specialized treatment. At the health-system level, standardized referral pathways could help community hospitals identify these rare tumors promptly and facilitate timely transfer of care when appropriate. Stronger coordination between community physicians and tertiary centers may also reduce unnecessary travel, as not every component of care must occur at the referral center. Complex surgery, radiation planning, and multidisciplinary treatment decisions may be centralized, while selected imaging surveillance, wound checks, rehabilitation, laboratory testing, and follow-up visits may be performed closer to the patient’s home.

Telemedicine and virtual multidisciplinary consultation may further strengthen these shared-care models by allowing community physicians to obtain specialist input before patients travel long distances and by supporting selected postoperative or surveillance visits. Timely evaluation at experienced centers may be particularly important given prior evidence associating specialized care with improved outcomes and our finding that receipt of surgery was associated with lower mortality in the adjusted survival models [7,8]. However, because county-level income is an ecological measure, it should not be used to infer an individual patient’s financial resources. Rather, the observed income gradient supports direct assessment of social and financial barriers so that supportive interventions can be targeted to the patients who need them, regardless of whether they live in a rural or urban area.

This study has several strengths. Because it uses a population-based registry design, it limits the referral and institutional selection bias inherent to single-center studies and captures patients treated across many institutions. All four histologic subtypes, chordoma, chondrosarcoma, osteosarcoma, and Ewing sarcoma, were analyzed together with histology as a covariate so that geographic and socioeconomic estimates would not be confounded by differences in subtype distribution between groups, a limitation of some of the single-histology studies that preceded it. By analyzing the mobile spine and sacropelvis together, the study reflects the anatomic region in which these tumors actually present and the surgical challenge they pose, rather than dividing the cohort along a boundary of limited clinical meaning. Lastly, rurality and county-level income were entered into the same adjusted models alongside stage, histology, and treatment. This allows geographic and socioeconomic disadvantage to be compared directly against each other rather than examined in isolation, as many prior studies in this area have done, which has made their conflicting results difficult to reconcile.

The study has limitations inherent to the retrospective analysis of the SEER registry. It is subject to unmeasured and residual confounding due to being an observational analysis. This has relevance to the association between surgery and lower mortality, as the patients who were selected for surgery may have likely differed in disease severity, resectability, and comorbidities. Cause of death may be misclassified, leading to possible bias in survival estimates. The registry does not adequately capture other clinically relevant characteristics, such as tumor size and histologic grade, which influence prognosis. Changes in classification, diagnostic criteria, and treatment over the study period may have influenced the observed outcomes.

The SEER county classification system is also limited due to measuring rurality by the geographical adjacency to a metropolitan area and population size. Applying county-level attributes to individual patients risks ecological fallacy and oversimplification of the individual-level risks. County-based rurality cannot fully act as a proxy for access to healthcare services, because Rural-Urban Continuum Codes are unable to measure the distance or time a patient must travel to reach care, facility volume and specialization, or whether referral or multidisciplinary care was provided. County-level median household income is similarly an ecological measurement and does not represent a patient’s individual income.

Anatomic site coding creates further limitations. ICD-O-3 assigns the sacrum and coccyx bones the same topography code as the pelvic bones (C41.4) [16]. Because many primary spinal tumors arise within the sacrum, the code was necessary to include within the cohort. Because this code represents a combination of sacrococcygeal and pelvic primary tumors, it may bias estimates of site distribution and outcomes.

Because surgery and radiotherapy were coded as fixed baseline covariates, due to sparsity in time to treatment availability, reflecting first course of treatment rather than as time-varying exposures, the observed association between surgery and lower mortality may be partly attributable to immortal time bias, whereby patients who survived long enough to receive treatment were inherently guaranteed to survive to that point.

Year or era of diagnosis was not included as a covariate in the multivariable models. This decision reflects several considerations: the rarity of these tumors already limited statistical power for subgroup analyses, and further stratification by diagnosis era risked sparse cell sizes across combinations of histology, extension, and rurality; the primary aim of this study was to characterize the relationship between geographic and socioeconomic exposures and survival rather than to model secular trends in treatment effectiveness; and the multivariable model already required several time-interaction terms to satisfy the proportional hazards assumption, such that an additional temporal covariate risked further model complexity without a clear gain in addressing the primary study question. Nonetheless, this study spans a 24-year period (2000-2023), during which diagnostic imaging, staging accuracy, surgical technique, systemic therapy, radiotherapy delivery, and supportive care for these malignancies evolved substantially. As a result, the reported associations, particularly the effect estimates for surgery, radiotherapy, and tumor extension, reflect an average effect across a period of considerable practice change and may not generalize uniformly to patients diagnosed at either end of the study period. If treatment effectiveness or the completeness of staging information improved over calendar time, this could bias the treatment and extension HRs in an unpredictable direction, and may also contribute to the non-proportional hazards observed for tumor extension and classification, as patients diagnosed later may have experienced systematically different hazard trajectories than those diagnosed earlier. Because rurality and county-level income were the primary exposures of interest, it is also possible that the accessibility gap between rural and urban or high- and low-income counties narrowed or widened over the study period; if such secular changes in disparity occurred, an unmodeled calendar time effect could mask or partially confound the associations reported here. Future studies incorporating year or era of diagnosis as a covariate, ideally with formal assessment of its interaction with treatment and outcome, would help clarify whether the associations reported here are consistent across the study period.

Lastly, the Fine-Gray sensitivity analysis was limited by non-proportional subdistribution hazards for several covariates, which precluded reporting adjusted HRs for the full model; only the exposures of primary interest, for which proportionality held, are reported.

## Conclusions

In this population-based retrospective cohort, greater tumor extension was independently associated with higher mortality, while receipt of surgical treatment was associated with lower overall and cancer-specific mortality. Histologic subtype also remained an important prognostic factor, which highlights the influence of tumor biology and disease burden on survival. Beyond purely biological factors, mortality had an independently graded association with county-level median household income, whereas rural residence was not significantly associated with mortality. As rurality and low county income were closely overlapped, these outcomes may be affected by socioeconomic differences rather than solely geographic area. Rural-urban classification alone may inadequately identify vulnerable patients and the individual-level barriers to treatment.
